# Willingness to accept a second COVID-19 vaccination booster dose among healthcare workers in Italy

**DOI:** 10.3389/fpubh.2022.1051035

**Published:** 2022-12-09

**Authors:** Giorgia Della Polla, Grazia Miraglia del Giudice, Lucio Folcarelli, Annalisa Napoli, Italo Francesco Angelillo, The Collaborative Working Group, Walter Longanella

**Affiliations:** ^1^Department of Public Health and Laboratory Services, Teaching Hospital, University of Campania “Luigi Vanvitelli”, Naples, Italy; ^2^Department of Experimental Medicine, University of Campania “Luigi Vanvitelli”, Naples, Italy

**Keywords:** COVID-19, HCWs, Italy, second booster dose, vaccination, willingness

## Abstract

**Background:**

The coronavirus disease 2019 (COVID-19) pandemic caused by severe acute respiratory syndrome coronavirus 2 (SARS-CoV-2) is evolving,the newly emerged Omicron variant being the dominant strain worldwide, and this has raised concerns about vaccine efficacy. The purposes of this survey were to examine the extent to which healthcare workers (HCWs) intend to receive a second booster dose of the COVID-19 vaccine and the factors that influence their willingness to accept it.

**Methods:**

The study was conducted among HCWs who were randomly selected from four public hospitals in the Campania region, Southern Italy.

**Results:**

A total of 496 HCWs answered the questionnaire (a response rate of 61.2%). Among the respondents, 20.8% indicated a score of 10, using a 10-point Likert-type scale, regarding the usefulness of a second COVID-19 vaccine booster dose. Physicians, HCWs who believed that COVID-19 was a severe disease, and those who have acquired information about the second booster dose from scientific journals were more likely to have this positive attitude. Slightly more than half of HCWs self-reported willingness to receive a second booster dose. Respondents who believe that HCWs are at higher risk of being infected by SARS-CoV-2, those who have a higher belief that COVID-19 is a severe disease, and those who have a higher belief that a second booster dose is useful were more willing to receive a second booster dose. The main reasons for those who had a positive intention were to protect their family members and patients, whereas, the main reasons for not getting vaccinated or for uncertainty were that the dose does not offer protection against the emerging variants and the fear of its side effects. HCWs of younger age, physicians, those who have a higher belief that a second booster dose is useful, and those who were willing to receive a second booster dose were more likely to recommend the booster dose to their patients.

**Conclusion:**

This study's findings highlight the necessity for designing and implementing educational interventions for improving second booster dose uptake and beliefs among HCWs and their capacity to recommend the vaccine to the patients.

## Introduction

The coronavirus disease 2019 (COVID-19) pandemic caused by severe acute respiratory syndrome coronavirus 2 (SARS-CoV-2) has generated more than half a billion confirmed cases and almost 6.5 million deaths around the world (1), including over 23.5 million and 179,000 people in Italy by 31 October 2022 (2). Several measures have been implemented to contain and prevent the spread of the disease, such as hand hygiene, social distancing, wearing a mask, and vaccination. However, SARS-CoV-2 is continuously evolving with the newly emerged Omicron variant being the dominant strain worldwide (3, 4), and this has raised concerns about vaccine efficacy. In Italy, on 11 July 2022, the Ministry of Health for this evolving scenario recommended an additional second booster dose or “fourth dose” of the currently available mRNA COVID-19 anti-Omicron variant vaccines, at least 4 months (120 days) after the first booster dose or the last post-booster infection (date of the positive test), to adults aged 60 years and above and individuals aged 12 years and above with concomitant/preexisting conditions (5). As of 19 September 2022, less than one-fourth of those eligible had received this second booster dose (6). Healthcare workers (HCWs), one of the most affected groups (7–9), have not been included, although, from 27 November 2021, the Italian government made vaccination with three doses mandatory for this group but this does not include the second booster dose (10). Moreover, HCWs also play an important role in transmitting the virus to their patients while providing care.

From this point of view, it is, therefore, extremely important and crucial to understand and assess HCWs' willingness to have the second booster dose of the COVID-19 vaccine; no literature is available on this topic. Therefore, the purposes of this present survey were to examine the extent to which a large sample of HCWs in Italy intends to receive a second booster dose of the COVID-19 vaccine and the factors that influence their willingness for accepting it.

## Materials and methods

### Setting and study population

The study was carried out from 12 July to 9 September 2022. The source population included all 4,000 HCWs who worked in different wards in four randomly selected public hospitals, one teaching and three nonteaching, located in the Campania region, Southern Italy. The sample for the present study included 496 HCWs who had been selected by a simple random sampling technique. A sample size of 384 HCWs was estimated assuming that 50% of the study population would intend to receive a second booster of the COVID-19 vaccine, 95% confidence interval, and a margin of error of 5%.

### Data collection

Initially, the research team asked for permission from the health director of each hospital to conduct the study. After the approval, the team identified in each ward an HCW to distribute the questionnaire to the HCWs who were randomly selected from the list of those present at that moment in each ward and to collect the filled questionnaires within an envelope to maintain anonymity and to return the envelope. The questionnaire contained a brief introduction about the objectives, procedure, confidentiality, and anonymity of the survey, that the participation was voluntary, that the information provided will be used only for research purposes, and that the participant was able to withdraw at any moment. HCWs gave their informed consent to participate by handing in the questionnaire. The participants received no incentive to complete the questionnaire.

### Survey development

All data were collected through a self-administered questionnaire adopted and modified from previously published studies of the research group (11–20). The questionnaire required 5–10 min to complete and capture the following information: a) sociodemographic, general, and professional characteristics (14 questions), including gender, age, relationship status, degree of education, professional role, duration of employment in the healthcare profession, area of working activity, self-rated health status, and previous COVID-19 infection; b) source(s) from which they receive information related to the second booster dose and whether they would like to get additional information (2 questions); and c) attitudes and behaviors (7 questions). The first comprised 5 items concerning attitudes toward COVID-19 and the second booster dose, using a 10-point Likert-type scale with a response format ranging from 1 = not at all to 10 = a great deal and a 5-point Liker-type scale ranging from 1 = strongly disagree to 5 = strongly agree, assessing whether the responder had been/had not been vaccinated with a second booster dose and the related reason(s). Those unvaccinated were asked to indicate whether they were willing or unwilling to receive a second booster dose and the underlying reasons in favor of or against receiving this vaccination. The survey was first piloted and tested by the research team to assess the feasibility and acceptability of the questions.

Ethical approval of the study protocol and questionnaire was received from the Ethics Committee of the Teaching Hospital of the University of Campania “Luigi Vanvitelli.”

### Statistical analysis

All statistical analyses were conducted using the software STATA version 15.1. Descriptive statistics were used with frequency, mean, and standard deviation to describe the principal characteristics of the participants, as well as behavior and attitude toward having a second COVID-19 booster dose. Multiple logistic regression models were built using the strategy suggested by Hosmer et al. (21). Each variable was examined by univariate analysis, using the chi-square test and Student's *t*-test, to evaluate predictors of the different outcomes of interest. Only those variables with a *p* < 0.25 in the univariate analysis were entered into three multivariate logistic regression models to assess associations between the main dependent variables and the several independent variables. Then, multivariate logistic regression analysis with backward elimination of any variable that did not contribute to the model on the grounds of the Likelihood Ratio test (cut-off at *p* = 0.05) was performed. Variables whose exclusion altered the coefficient of the remaining variables were kept in the model. Backward stepwise selection has been used with a threshold of *p* = 0.2 and *p* = 0.4, respectively, for the entry or removal of the variables from the final models. Odds ratios (OR) and their corresponding 95% confidence intervals (CI) were calculated in the models. Three outcomes of interest have been identified: a) belief that a second booster dose of the COVID-19 vaccine was useful (1–9 = 0; 10 = 1) (Model 1); b) willingness to receive a second booster dose of the COVID-19 vaccine (no/do not know = 0; yes = 1) (Model 2); c) recommendation of a second booster dose of the COVID-19 vaccine to the patients (no = 0; yes = 1) (Model 3). The following potential determinants were included in all models: gender (female = 0; male = 1); age, in years (continuous); marital status (unmarried/separated/divorced/widowed = 0; married/cohabitant = 1); physicians (no = 0; yes = 1); length of practice, in years (less than three = 0; at least three = 1); having worked in a COVID-19 ward (no = 0; yes = 1); having underlying at least one chronic medical condition (no = 0; yes = 1); having been tested positive for COVID-19 (no = 0; yes = 1); at least one family member/colleague/friend who had been tested positive for COVID-19 (no = 0; yes = 1); perceived risk of getting infected with SARS-CoV-2 during the working activity (1–9 = 0; 10 = 1); belief that COVID-19 is a serious disease (1–9 = 0; 10 = 1); belief that HCWs are at a higher risk of being infected by SARS-CoV-2 (strongly disagree/disagree/undecided = 0; agree/strongly agree = 1); scientific journals as source of information about the second booster dose of the COVID-19 vaccine (no = 0; yes = 1); and needing additional information regarding the second booster dose of the COVID-19 vaccine (no = 0; yes = 1). Moreover, the variables belief that the second booster dose of the COVID-19 vaccine was useful (1–9 = 0; 10 = 1) and belief that the second booster dose of the COVID-19 vaccine was effective (1–9 = 0; 10 = 1) were included in Models 2 and 3; and the variable willingness to receive the second booster dose of the COVID-19 vaccine (no/undecided = 0; yes = 1) was included in Model 3. For all analyses, two-tailed tests were used and statistical significance was determined with a *p*-value equal to or less than 0.05.

## Results

A total of 496 HCWs, out of the 810 selected, answered the questionnaire with a response rate of 61.2%. The main sociodemographic, general, and professional characteristics of the respondents are summarized in Table 1. The average age was 42.3 years, almost two-thirds were female participants, more than half were nurses/midwives, two-thirds worked in medical wards, almost one-third have had working experience in a COVID-19 ward, the mean length of working activity was 13.7 years, only 15.1% self-identified as having a chronic medical condition, more than half have had COVID-19, almost all had a family member/colleague/friend who tested positive for COVID-19, and only 25 of the 52 eligible has been vaccinated with a second booster dose.

**Table 1:** Main sociodemographic and general characteristics of the sample.

**Characteristics**	***N***	**%**
**Age, years (496)**	42.3 ± 12.4 (22-78)^*^	
**Gender (493)**		
Male	181	36.7
Female	312	63.3
**Marital status (446)**		
Married/cohabited with a partner	272	61.0
Unmarried/separated/divorced/widowed	174	39.0
**Professional role (493)**		
Physician	185	37.5
Nurse/Midwife	257	52.1
Other	51	10.4
**Length of practice, in years (458)**	13.7 ± 11.7 (1-41)^*^	
Less than three	106	23.1
At least three	352	76.9
**Current working area (496)**		
Medical	369	74.3
Surgical	83	16.8
COVID-19 ward	44	8.9
**Having ever worked in a COVID-19 ward (493)**		
No	348	70.6
Yes	145	29.4
**At least one chronic medical condition (495)**		
No	420	84.9
Yes	75	15.1
**Having been vaccinated against COVID-19 (490)**		
No	1	0.2
**Yes**	489	99.8
With less than three doses	25	5.1
With at least three doses	464	94.9
**Having been vaccinated against COVID-19 with a second booster dose (among eligible) (52)**		
No	27	51.9
Yes	25	48.1
**Having been tested positive for COVID-19 (493)**		
No	210	42.6
**Yes**	283	57.4
Once (281)	244	86.8
More than once (281)	37	13.2
**At least one family member/colleague/friend who tested positive for COVID-19 (476)**		
No	27	5.7
Yes	449	94.3
**Having been vaccinated against influenza in the previous year (496)**		
No	331	66.7
Yes	165	33.3

In brackets is reported the number of respondents for each variable.

* Mean ± Standard deviation (range).

The results regarding the attitudes, measured on a 10-point Likert-type scale, showed that the mean scores of the respondent's belief that COVID-19 was a severe disease and whether they feel at risk of being infected by SARS-CoV-2 during the working activity were 7.4 and 6.8, respectively, with 19.6% believing themselves to be at an elevated risk (as by indicated a value of 10). The mean scores regarding the usefulness and efficacy of a second booster dose of the COVID-19 vaccine were 6.7 and 6, respectively, with only 20.8% and 16.4% of participants who had indicated a score of 10. Table 2 presents the results from the three multivariate logistic regression models examining the relationship between several variables and the different outcomes of interest. The first model showed that a score of 10 regarding the usefulness of a second booster dose of the COVID-19 vaccine was more likely to be observed in physicians (OR: 1.99, 95% CI: 1.14–3.46), in those who have a higher belief that COVID-19 was a severe disease (OR: 4.47, 95% CI: 2.39–8.37), and in those who have acquired information about the second booster dose of the COVID-19 vaccine from scientific journals (OR: 2.24, 95% CI: 1.31–3.85).

**Table 2:** Results of the multivariate logistic regression analysis showing determinants of the different outcomes of interest.

**Variable**	**OR**	**SE**	**95% CI**	***p***
**Model 1**. Belief that a second booster dose of the COVID-19 vaccine was useful (Sample size=491)				
Log likelihood = −201.31, χ^2^ = 62.76 (6 df), *p* < 0.0001				
Higher perception of the severity of COVID-19	4.47	1.43	2.39-8.37	<0.001
Having received information on a second booster dose of the COVID-19 vaccine from scientific journals	2.24	0.62	1.31-3.85	0.004
Physicians	1.99	0.56	1.14-3.46	0.015
Higher perceived risk of being infected by SARS-CoV-2 during the working activity	1.86	0.61	0.98-3.53	0.058
Not having been tested positive for COVID-19	0.63	0.16	0.38-1.04	0.072
Knowing at least one family member/colleague/friend who tested positive for COVID-19	2.96	2.31	0.64-13.71	0.165
**Model 2**. Willingness to receive a second booster dose of the COVID-19 vaccine (Sample size=431)				
Log likelihood = −240.39, χ^2^ = 45.16 (8 df), *p* < 0.0001				
Higher perception of the utility of a second booster dose of the COVID-19 vaccine	2.71	0.85	1.47-5.01	0.001
Believing that HCWs are at high risk of being infected by SARS-CoV-2	1.89	0.51	1.13-3.19	0.016
Higher perception of the severity of COVID-19	2.01	0.65	1.06-3.77	0.031
Having received information on a second booster dose of the COVID-19 vaccine from scientific journals	1.54	0.37	0.96-2.47	0.072
No need to receive additional information about a second booster dose of the COVID-19 vaccine	0.74	0.17	0.47-1.16	0.19
Less than three years of practice	0.69	0.19	0.39-1.21	0.2
Physicians	1.32	0.34	0.79-2.21	0.282
Not having any chronic medical condition	0.73	0.24	0.39-1.39	0.344
**Model 3**. HCWs who recommend a second booster dose of the COVID-19 vaccine to their patients (Sample size=462)				
Log likelihood = −148.73, χ^2^ = 102.35 (7 df), *p* < 0.0001				
Willingness to receive a second booster dose of the COVID-19 vaccine	10.21	3.52	5.19-20.06	<0.001
Higher perception of the utility of a second booster dose of the COVID-19 vaccine	6.78	4.43	1.88-24.43	0.003
Younger	0.96	0.01	0.93-0.99	0.005
Physicians	2.45	0.88	1.20-4.97	0.013
At least three years of practice	1.96	0.85	0.84-4.58	0.12
Higher perceived risk of being infected by SARS-CoV-2 during the working activity	1.86	0.78	0.82-4.25	0.14
Not having received information on a second booster dose of the COVID-19 vaccine from scientific journals	0.73	0.24	0.38-1.40	0.173

Among those respondents who had not had the second booster dose of the COVID-19 vaccine, 52.6% self-reported a willingness to receive it, and 25.1% and 22.3% indicated that they had “no intention” or showed “uncertainty.” The main self-reported reasons for those who had a positive intention were to protect their family members (49.6%) and their patients (42.9%) and the fear of acquiring the infection (37.6%). The main reasons for not getting vaccinated or for uncertainty, however, were that the dose does not offer protection against the emerging variants (54.6%) and the fear of its side effects (27%). Three variables were found to be associated with the HCWs' willingness to receive a second booster dose in the multivariate logistic regression analysis. Respondents who believed that HCWs are at higher risk of being infected by SARS-CoV-2 (OR: 1.89, 95% CI: 1.13–3.19), those who have a higher belief that COVID-19 was a severe disease (OR: 2.01, 95% CI: 1.06–3.77), and those who have a higher belief that a second booster dose is useful (OR: 2.71, 95% CI: 1.47–5.01) were more willing to receive a second booster dose of the COVID-19 vaccine (Model 2 in Table 2). A total of 75.3% of HCWs recommend the booster dose to their patients, whereas among those who did not recommend it, 83.6% were unwilling to make the recommendation. HCWs were more likely to recommend the booster dose to the patients if they were younger (OR: 0.96, 95% CI: 0.93–0.99), physicians (OR: 2.45, 95% CI: 1.20–4.97), have a higher belief that a second booster dose is useful (OR: 6.78, 95% CI: 1.88–24.43), and if they were more willing to receive a second booster dose (OR: 10.21, 95% CI: 5.19–20.06) (Model 3 in Table 2).

Almost all HCWs had received information about the second COVID-19 booster dose (96.6%). The internet (51.8%), mass media (48.6%), scientific meetings (48.2%), and scientific journals (41.5%) were indicated as primary sources for this information, followed by social networks (26.7%). More than one-third of the respondents expressed an interest in acquiring additional information about the second booster dose (36.3%).

## Discussion

To our knowledge, this is the largest survey of HCWs' willingness to have a second booster dose of the COVID-19 vaccine and the factors associated with this decision conducted in Italy. The major findings can be summarized in the following five points. First, slightly more than 50% of the sample would accept a second booster dose of the COVID-19 vaccine. Second, the main reasons behind the willingness to have a second booster dose were to protect family members and patients. Third, the main reasons for the intention to not receive or uncertainty toward the second booster dose were the belief that it does not offer protection against the emerging variants and the fear of side effects. Fourth, scientific meetings and journals were among the primary sources of information on the second booster dose. Fifth, several determinants have been observed to be significantly associated with the different outcomes of interest.

Overall, the present survey revealed that only 52.6% of respondents self-reported a willingness to receive a second booster dose. Though it is only mandatory for HCWs to have the first COVID-19 booster dose, it was nonetheless a striking and unexpected finding that very few (48.1%) eligible HCWs had received a second booster dose. The prevalence of this willingness was lower than the values observed among HCWs in Saudi Arabia (55.3%) (22), Czechia (71.3%) (23), and China (87%) (24). A surprising finding was that this value was also considerably lower than those in the general population in India (59.1%) (25), the Middle East and North Africa Region (60.2%) (26), China (91.1%) (27), Japan (97.8%) (28), university students and staff in Italy (85.7%) (15), and the United States (96.2%) (29). The finding of the present study is of great concern because HCWs have a higher risk of infection with SARS-CoV-2 than the general population; in Italy, since the beginning of the pandemic as of September 2021, there have been 3,970 deaths among HCWs out of a total of 124,000 (30). This alarming picture has had an important impact on the healthcare delivery system, with the difficulty in maintaining levels of care and in responding to the population's needs. Therefore, it is important to increase willingness and uptake of a second COVID-19 booster dose since it has been reported in the literature that vaccinated HCWs, as other groups of individuals, have a considerable influence on their patient's intention to get vaccinated or more likely to deliver the vaccinations (31–34).

This study highlighted that the protection of their family members and patients and the fear of acquiring the infection were the most frequent reasons for the willingness to receive a second booster dose of the COVID-19 vaccine. These findings are consistent with other recent similar research studies (35–38). A possible explanation for the protection of the family is that household transmission has been observed as one of the most common primary routes of SARS-CoV-2 transmission (39–43). Therefore, vaccines and boosters are the best primary interventions for preventing SARS-CoV-2 transmission since, in the household, it is not easy to maintain social distancing, avoid close contacts, and wear masks. Moreover, among those HCWs who did not intend to receive the second booster dose or were uncertain, concerns about the safety and effectiveness of the vaccine against the emerging variants were the most common reasons. Previous studies among different samples and geographic areas have linked these reasons with hesitancy or unwillingness to get vaccinated against COVID-19 (44–48). Addressing these concerns is of crucial importance to improve the uptake of a second booster dose also at the population level through evidence-based messages considering the pivotal role of the HCWs in community health.

The results of the multivariate logistic regression analysis showed that several factors were significant predictors of attitude, vaccine willingness, and vaccine recommendation. Of the several sociodemographic and professional characteristics, only age and professional role were associated with the outcomes of interest. Indeed, physicians indicated a higher score regarding the usefulness of a second booster dose of the COVID-19 vaccine, and as, those younger, they were more likely to recommend this booster dose to the patients and more willing to receive it. Moreover, three variables related to the respondents' attitudes have had a significant impact. HCWs who believed that COVID-19 was a serious disease and who believed that they are at higher risk of being infected by SARS-CoV-2 were more likely to believe that the second booster dose is useful and more willing to receive the booster dose, and HCWs who believed that the second booster dose is useful and who were willing to receive it were more likely to recommend the booster dose to the patients. Therefore, it is extremely important that the HCWs should be aware of the vaccine's effectiveness in preventing SARS-CoV-2 infection and to improve their attitudes as an effective way to enhance HCWs' willingness to be vaccinated with a second booster dose of the COVID-19 vaccine or to recommend it. Some of these associations have been observed in a previous investigation (49).

This present survey showed that almost all HCWs had received information related to a second booster dose of the COVID-19 vaccine, with scientific meetings and journals being two of the most trusted sources. It is important to highlight that scientific journals have a significant effect on the higher belief regarding the usefulness of a second booster dose. This finding confirms that these sources are an important factor in the vaccination process and decision. Indeed, this association is in accordance with previous studies that showed that scientific journals played a significant role in determining a higher level of knowledge, a more positive attitude, an increase in the willingness to receive a vaccine, and a higher vaccination coverage among those who have acquired information from these sources (17, 18). Moreover, it should also be noted that mass media, social media, and the internet were also accepted by many HCWs. However, these sources need to be carefully used because evidence indicated that there is the possibility of the spread of untrue and negative information, resulting in worry about the COVID-19 vaccination, lower coverage, and higher hesitancy (50, 51). It is interesting to observe that a systematic review of the reviews regarding infodemics and health misinformation indicated that social media has been increasingly propagating poor quality health-related information during pandemics and health emergencies (52).

The results from the present survey should also be considered with some potential methodological limitations. First, as in all cross-sectional studies, no causal relationships between the independent variables and the different outcomes of interest can be established. Second, the survey was administered to HCWs in a single geographic area, and therefore, the findings may not necessarily apply to other areas of Italy. Third, a self-reporting questionnaire may have introduced social desirability bias and the surveyed HCWs may tend to have more positive attitudes that lead to an overestimation of their intention to have a second booster dose of the COVID-19 vaccine. However, an anonymous questionnaire has been used to reduce this bias. Despite these limitations, this study was the first to assess the willingness to have a second booster dose among HCWs in Italy, and it thus provides an important picture with important implications for health policymakers.

In conclusion, this survey reveals a low willingness to receive a second booster dose, the facilitators and barriers influencing this willingness, and the factors associated with this choice. The findings have important implications and highlight the necessity for designing and implementing targeted education interventions for improving the second booster dose of the COVID-19 vaccine uptake among HCWs and their capacity to recommend the vaccine to the patients. In the future, investigations are expected to quantify the coverage level in HCWs and to evaluate whether they can promote this vaccination with special attention toward more vulnerable people.

## The collaborative working group

Walter Longanella (Health Direction, San Giovanni di Dio Ruggi D'Aragona Hospital, Largo Città Ippocrate, 84131 Salerno, Italy), Mario Massimo Mensorio (Health Direction, Sant'Anna e San Sebastiano Hospital, Via Ferdinando Palasciano, 81100 Caserta, Italy), Federica Cantore (Health Direction, San Giuseppe Moscati Hospital, Contrada Amoretta, 03100 Avellino, Italy).

## Data availability statement

The raw data supporting the conclusions of this article will be made available by the authors, without undue reservation.

## Ethics statement

The studies involving human participants were reviewed and approved by Teaching Hospital of the University of Campania Luigi Vanvitelli. The patients/participants provided their written informed consent to participate in this study.

## Author contributions

GDP, GMdG, LF, and AN participated in the conception and design of the study and contributed to the data collection, data analysis, and interpretation. IFA the principal investigator, designed the study, was responsible for the statistical analysis and interpretation, drafted and wrote the article. All authors have read and approved the final version of the article and agree to be accountable for all aspects of the work.
